# Monitoring changes in mixed venous oxygen saturation using photoplethysmography on the sternum – a proof of concept study

**DOI:** 10.1007/s11517-026-03648-3

**Published:** 2026-07-30

**Authors:** Erik Näslund, Gunnar Strandberg, Jacob Karlsson, Stephanie Franzén, Robert Frithiof

**Affiliations:** 1 Section of Anaesthesia and Intensive Care, Department of Surgical Sciences, Uppsala University, Uppsala, Sweden; 2 Centre for Research and Development, Uppsala University, Region Gävleborg, Gävle, Sweden; 3 Department of Physiology and Pharmacology, FYFA), C3, Per-Arne Lönnqvist group – Section of Anesthesiology and Intensive Care, Karolinska Institute, Stockholm, Sweden; 4 Department of Medicine, Section Nephrology, Cardio-Renal Physiology and Medicine, University of Alabama at Birmingham, Birmingham, AL USA

**Keywords:** Sternum, Photoplethysmography, Mixed venous oxygen saturation, Mixed linear models, Concordance rate

## Abstract

Mixed venous oxygen saturation (SvO_2_) is a valuable indicator of the balance between oxygen delivery and consumption in the body. However, the invasiveness and risk of complications limit its use. This study aimed to test the feasibility of continuously monitoring variations in SvO_2_ from photoplethysmography (PPG) measurements of sternal intramedullary oxygen saturation. In an animal model, including anesthetized pigs (*n* = 15, mean 33.1 (SD 2.0) kg), alterations in SvO_2_ were instituted by separate sessions: incremental decrease in arterial oxygen saturation using hypoxic gas mixtures (N_2_/air), and hemorrhage of 20% plus 20% of estimated blood volume. Estimations of non-invasive sternal oxygen saturation (nSsO_2_) were derived from calibrating PPG measurements against SvO_2_ sampled from the pulmonary artery. Individual linear correlations were found between SvO_2_ and PPG measurements. nSsO_2_ and SvO_2_ co-varied, except in severe hypovolemia. A concordance rate between nSsO_2_ and SvO_2_ was determined to 87% (95% CI 79.1–95.0). In this study, we demonstrated linear correlations between SvO_2_ and PPG-measurements on the sternum, enabling estimation of nSsO_2_, which trends with changes in SvO_2_. The results support further technical development of non-invasive monitoring of SvO_2_ from sternal measurements.

**Graphical abstract:**

Non-invasive sternal oxygen saturation (nSsO_2_) is derived from photoplethysmographic measurements of sternal intramedullary blood flow (SQV), correlated against mixed venous oxygen saturation (SvO_2_). The performance of nSsO_2_ to detect changes in SvO_2_ is subsequent analyzed with a concordance analysis.

**Figure:**
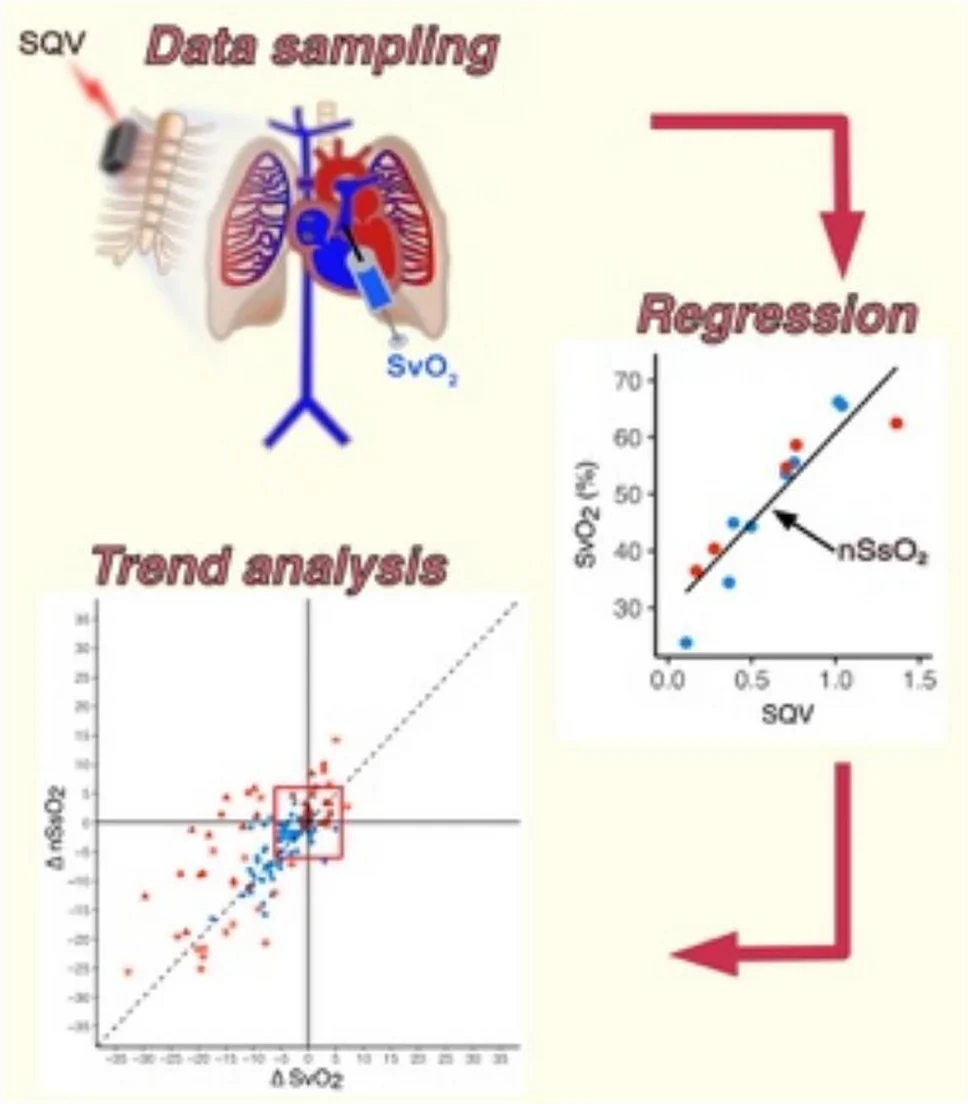

## Introduction

Mixed venous oxygen saturation (SvO_2_) is measured from the pulmonary artery and depends on whole-body oxygen delivery and consumption. SvO_2_ is therefore of clinical interest to monitor in certain situations, such as circulatory or septic shock, guidance for optimization of fluid balance or treatment with vasoactive drugs, or after major heart surgery. However, SvO_2_ is infrequently used due to its inaccessibility and need for advanced cannulation of intrathoracic vessels, increasing the risk for complications affecting morbidity and sometimes even mortality [1, 2]. In addition, the equipment necessary for continuous SvO_2_-monitoring is not always available at standard intensive care units. Therefore, a possibility of non- or semi-invasive means of measuring or estimating SvO_2_ is of great interest. Unfortunately, currently available alternative methods for the continuous assessment of SvO_2_ are either not representative of a true SvO_2_ value or not yet fully validated in the clinical context (e.g., near-infrared spectroscopy (NIRS) and capnodynamics) [3–8].

The oxygen saturation in the blood is defined as the proportion of occupied hemoglobin binding sites by oxygen molecules, expressed as a percentage of the total hemoglobin binding capacity [9].

There is a distinction between how the arterial versus venous oxygen saturation is formed. The arterial oxygen saturation is primarily dependent on the partial pressure of oxygen. In contrast, the venous oxygen saturation is dependent on the balance of oxygen supply and demand, which may vary among locations as different tissue beds have varying metabolic needs [9].

We have previously proposed a novel method for non-invasive measurement of sternal intramedullary oxygen saturation using a wireless photoplethysmography (PPG) sternal prototype probe [10, 11]. The sternum has a mixed arteriovenous blood flow [12, 13]. To investigate how sternal intramedullary oxygen saturation (SsO_2_) responds to different causes of decreased oxygen delivery, we previously conducted a study using a porcine model of both hypoxemia and hypovolemia. From that study, we concluded that SsO_2_, resembled SvO_2_ more closely than arterial oxygen saturation. Additionally, non-invasive PPG measurements over the sternum exhibited the same pattern of changes as SsO_2_ [11]. However, measurements in that study were obtained only at baseline and during a defined provocation, while the changes occurring between these time points were not assessed. Even though SsO_2_ differs from SvO_2_, based on previous results, we hypothesized that changes in SsO_2_ co-varies with changes in SvO_2_. Such a relationship could enable a simple method for detecting a change in the body's utilization of oxygen.

The aim of the current study was to determine whether non-invasive PPG measurements of SsO_2_ could enable continuous monitoring of variations in SvO_2_. To achieve this, our first objective was to calibrate non-invasive PPG measurements from the sternum against blood gas measurements obtained from mixed venous blood in the pulmonary artery. From this calibration process, we estimated a variable, we refer to as non-invasive SsO_2_ (nSsO_2_). The second objective was to assess how well nSsO_2_ could detect concomitant changes in SvO_2_ as assessed by the reference method—blood gas analysis from the pulmonary artery.

## Methods

### Study subjects

The Uppsala Animal Ethics Committee, Uppsala, Sweden, approved the study (5.8.18–02325/2019, date of approval 2019–03–29). All animals were treated according to the Swedish Board of Agriculture guidelines and complied with the European Convention for the Protection of Vertebrate Animals used for Experimental and other Scientific Purposes (Council of Europe No 123, Strasbourg 1985) and the Animal Research: Reporting of In Vivo Experiments (ARRIVE 2.0) guidelines.

The study included 15 Norwegian Landrace breed/Hampshire/Yorkshire pigs of both sexes aged 3–4 months. All pigs were acquired from a local breeder, where they were housed in groups and raised according to the farmer’s standard protocol.

### Anesthesia and preparation

The pigs arrived at the laboratory at 8.00 am local time. Upon arrival, all animals were calm and normothermic. One animal per day was included in the study. All animals were anesthetized with an intramuscular injection of 6 mg kg^−1^ tilétamine-zolezepam (Zoletil, Virbac, Denmark) and 2.2 mg kg^−1^ xylazine (Rompun, Elanco, Denmark). A bolus dose of fentanyl 100 µg was administered intravenously after the establishment of peripheral venous access in an ear vein. The animals were placed in a supine position, tracheotomized, and mechanically ventilated in a volume-controlled mode to reach tidal volume 7 mL kg^−1^, respiratory rate 25 min^−1^, fraction of inspired oxygen (F_I_O_2_) 0.4, and positive end-expiratory pressure (PEEP) 5 cmH_2_O (Servo-I ventilator, Maquet Critical Care, Solna, Sweden) intending to maintain normocapnia.

Maintenance of general anesthesia was performed with an intravenous infusion of propofol 10 mg mL ^−1^ (10 mg kg^−1^ h^−1^) and fentanyl (30 μg kg^−1^ h^−1^). Rocuronium bromide (Esmeron, Merck & Co. Inc., Kenilworth, NJ, USA) was given 0.25 mg kg^−1^ h^−1^ to reduce motion artifacts. All animals were given a fluid bolus of 20 mL kg^−1^ during the first hour, and after that, the infusion rate was lowered to 10 mL kg^−1^ h^−1^ (1:1 ratio of Ringer's Acetate + buffered solution of Glucose 2.5 mg mL^−1^).

The animals were surgically catheterized with a 3-lumen central venous catheter inserted through a right-sided cervical vein, and forwarded to the central vena cava, along with a balloon-tipped pulmonary artery catheter (7.5F Swan-Ganz, Edwards Lifesciences, Irvine, CA). On the left side of the neck, a central 7.5F PA-introducer was inserted in a cervical vein, enabling hemorrhage. An arterial catheter was placed in the femoral artery using an ultrasound-guided Seldinger technique.

After thoroughly cleansing the skin, the sternal probe was attached to the caudal and flat surface of the sternum using double adhesive tape. All animals were given 30 min to recover before the experimental protocol commenced.

### Protocol

**Fig. 1:**
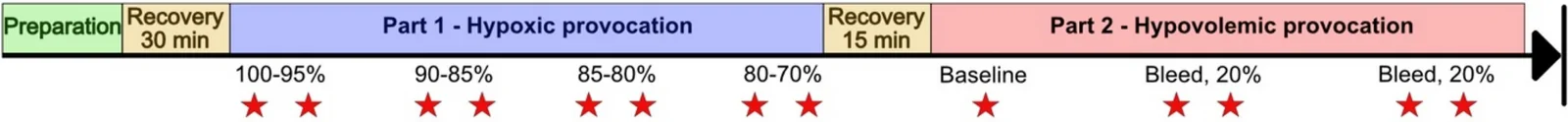
Schematic illustration of study protocol. For the hypoxic provocation, the percentages refer to intended oxygen saturation levels guided by peripheral pulse oximetry. The stars denote the time points for gathering sternal probe readings along with mixed venous blood gas sampling

The protocol consisted of two sections investigating the performance of nSsO_2_ (Fig. 1). The first part was progressing systemic hypoxemia, and the second part, controlled hemorrhage.

Hypoxemia was induced by incrementally adding N_2_ to the inspired gas mixture in the ventilator. Peripheral pulse oximetry aided in producing stable oxygen saturation levels of 100–95%, 90–85%, 85–80% and 80–70%, respectively. Blood samples were simultaneously collected from both arterial and pulmonary artery catheters, on two separate occasions for each oxygen saturation level. The samplings were separated by a period of at least 30 s. At the end of the hypoxic maneuver, ventilator settings were reset, and approximately 15 min of rest ensued.

Hypovolemia was instituted by hemorrhage of 20% of the estimated circulating blood volume (70 mL kg^−1^). After approximately five minutes, a second bleeding of another 20% was done. Sampling of arterial and mixed venous blood, and measurement of circulatory parameters, including determination of cardiac output using thermal dilution, were performed at baseline, directly after completion of the bleeding, and again after five minutes for both steps of the bleedings.

All blood samples were analyzed on an ABL800 blood gas analyzer (Radiometer AS, Copenhagen, Denmark) calibrated for porcine blood. The equipment was located within the laboratory, which ensured a minimal time delay before analysis. Basic physiological parameters, such as heart rate and blood pressure were continuously monitored throughout the experiment.

Sufficient anesthetic depth and analgesic level were tested regularly throughout the experiment by monitoring circulatory status in combination with any present motor reflexes from a pain stimulus to the hoof. The experiments were conducted under general anesthesia. At the end of the study protocol, the animals were euthanized with a 20 mL bolus dose of KCl (150 mg mL^−1^), administered in a central vein, under continuous ECG monitoring to ensure the onset of asystole. Included ethical endpoints for cessation of the study protocol were hyperthermia (> 40 °C) at arrival to the laboratory, mean arterial pressure (MAP) < 40 mmHg despite treatment, pulmonary artery pressure > arterial pressure despite treatment, and arterial oxygen saturation < 85% despite treatment (besides hypoxic provocation).

### Sternal probe data

The sternal probe was developed by RespiHeart AB and was loaned to the investigators for the purpose of this study. The company had no involvement in study design, data collection, analysis, interpretation of results, or manuscript preparation. The methodological background of the sternum probe has been described in detail previously [10, 11, 14, 15]. In short, the sternum probe utilizes red (660 nm) and infrared (810 nm) lights in reflectance mode PPG. The separation distance of the LED lights and photodiode (22.5 mm) allows deep penetration of light [16]. The ratio of the DC-components from the PPG-signal is used to form the sternal probe quotient value (SQV), which is used to estimate oxygen saturation. As opposed to previously described [10, 11], this study utilized an updated version of the sternum prototype probe, which included new LED and photodiode components. Also, on startup, light intensity and gain were automatically adjusted to achieve the best possible signal quality. However, the signal enhancements hampered comparisons between individuals since SQV-constructs became specific, hence were data analyzed individually. The 100 Hz raw signal from the sternal probe was transferred via Bluetooth communication to an Android tablet using a signal acquisition program. Matlab (R2022b, Mathworks, Massachusetts, USA) was used to post-process data. The DC-component of the acquired PPG-signal was extracted by applying a second-order Butterworth filter with a cutoff frequency of 0.4 Hz. As opposed to previously described [10], in this study, we choose to form the SQV by dividing DC-components of red light by infrared to better conform to standard PPG-methodology. The SQV values, which were determined for all corresponding time points when blood gases were sampled, were calculated as a four-second SQV-mean, including approximately 400 data points.

### Statistics

Data distribution was assessed using the Shapiro-Wilks test and by visually examining histograms. Normally distributed data are presented as mean (standard deviation (SD)) or mean (95% Confidence interval (CI)) as appropriate. Non-normal data are presented as median (Inter quartile range (IQR)). Descriptive statistics (Table 1) are presented as median (IQR), using the Wilcoxon signed rank test to analyze for differences, regardless of statistical distribution.

**Table 1:** Physiologic variables measured at baseline and at the end of the different provocations

	Part 1 Hypoxic provocation			Part 2 Hypovolemic provocation		
Variables	Normoxia^*1*^	Hypoxemia^*1*^	*p*-value^*2*^	Normovolemia^*1*^	Hypovolemia^*1*^	*p*-value^*2*^
HR^a^ (beat min^−1^)	105 (92–109)	110 (106–115)	0.006	92 (87–101)	90 (82–107)	0.8
MAP^b^ (mmHg)	65 (62–68)	52 (49–55)	0.001	61 (58–71)	34 (31–38)	< 0.001
mPAP^c^ (mmHg)	15 (14–16)	29 (25–31)	0.002	17 (15–18)	12 (10–16)	0.002
Hb^d^ (g L^−1^)	92 (84–97)	91 (89–99)	> 0.9	86 (80–91)	72 (68–76)	0.002
Hct^e^ (%)	29 (26–30)	28 (28–31)	> 0.9	27 (25–28)	23 (21–24)	0.002
SaO_2_^f^ (%)	97 (97–98)	73 (69–74)	< 0.001	97 (96–97)	97 (97–97)	0.4
SvO_2_^g^ (%)	62 (55–65)	31 (25–36)	< 0.001	57 (52–62)	24 (20–28)	< 0.001
nSsO_2_^h^ (%)	56 (53–63)	33 (31–39)	< 0.001	51 (48–54)	36 (33–40)	< 0.001
CO^i^ (L min^−1^)				4.1 (3.8–4.4)	2.5 (1.9–2.8)	< 0.001

^1^Median (IQR), *N* = 14

^2^Wilcoxon signed rank test

^a^Heart rate, ^b^Mean arterial pressure, ^c^Mean pulmonary pressure, ^d^Hemoglobin, ^e^Hematocrit, ^f^Arterial oxygen saturation, ^g^Mixed venous oxygen saturation, ^h^Non-invasive sternal intramedullary oxygen saturation, ^i^Cardiac output

#### The relationship between SQV/nSsO_2_ and SvO_2_

Estimating nSsO_2_ was performed using a regression model that included SvO_2_ and SQV. To account for repeated measures within subjects and clustered data (sampling from the different provocations), a linear mixed effects model was used [17].

To determine individual nSsO_2_ values, the model included SQV and SvO_2_ from both provocations as fixed effects, along with slope and intercept as random effects. Any violation of model assumptions was tested with residuals vs fitted plots, and normal quantile–quantile (QQ)-plots.

Measures of the goodness of fit, such as the coefficient of determination (R^2^), describe the amount of variation explained by a model and are well established for linear regression models, but are not as easily adopted regarding linear mixed effects models, including random effect structure. For the mixed-effects model, we report both the conditional R^2^ (including fixed and random effects) and the marginal R^2^ (fixed effects only) [18]. Because the analysis was performed at the individual subject level, the random effects contributed substantially to the total variance. Therefore, our interpretation focuses on the model’s explanatory capacity, as captured by the conditional R^2^.

There is no straightforward method for obtaining subject-specific R^2^ estimates in a linear mixed-effects model. As a surrogate measure of the strength of the individual-level fit, R^2^ was calculated using ordinary linear regression on paired raw data of SvO_2_ and SQV for each subject.

#### Trend analysis between nSsO_2_ and SvO_2_

The ability of SQV to track changes in global level of oxygen balance compared to the reference method SvO_2_ was assessed by calculating the concordance rate, i.e., the percentage of data points moving in the same direction when comparing two different techniques [19]. Previously published data has shown an inherent precision of ± 7% for the reference method CO-oximetry SvO_2_ when using the same blood gas machine calibrated for porcine hemoglobin in the same laboratory [20]. This precision was then used to calculate the least significant change (LSC), i.e., the minimum change measured and recognized as a true change for the reference method [21]. LSC was used to determine the exclusion zone in the concordance analysis as previously described [8]. The LSC for CO-oximetry was found to be 9.5%. Based on this, changes in SvO_2_ of less than 9.5% (rounded up to 10%) of the mean of all SvO_2_ CO-oximetry recordings (corresponding to a minimal change in CO-oximetry SvO_2_ of 6 percentage points) were not regarded as true changes and excluded from the concordance analysis. A 95% CI for the concordance rate was also calculated as previously described [22].

A concordance rate of > 92% was considered a good result, based on clinical judgment and investigation of related monitoring methods [19, 20].

All statistical analysis and graphics were done using R Statistical Software (v4.3.1; R Core Team 2021). The mixed models were constructed using the lme4-package (v1.1.34; Bates et al., 2015) together with the lmerTest-package (v3.1.3; Kuznetsova, Brockhoff, and Christensen 2017) to approximate p-values using Satterwaith's method. A two-sided p-value of < 0.05 was considered statistically significant for all analyses.

## Results

The included animals (7 male/8 female) had a mean weight of 33.1 (SD 2.0) kg. One subject could not complete the protocol due to severe hemodynamic instability. The remaining 14 animals produced 182 paired observations between SvO_2_ and SQV. Five observations were excluded due to malfunction of the blood gas analyzer, thus generating a total of *n* = 177 paired measurements.

According to measured vital parameters, all pigs were in a stable physiological state before the hypoxic and hypovolemic provocations were commenced (Table 1). F_I_O_2_ required to reach the predefined oxygen saturation levels, measured by pulse oximetry, ranged between 0.4–0.12 (the latter was the lowest value the oxygen sensor in the ventilator could measure). Hypoxemia induced an expected marked drop in systemic arterial saturation, SvO_2,_ and nSsO_2_. Additional recorded data is displayed in Table 1.

During hemorrhage, the mean bleeding times were 5 min 1 s (SD 1 min 12 s) and 4 min 47 s (SD 1 min 43 s) for the first and second bleeding, respectively. SaO_2_ remained constant throughout the hypovolemic provocation, but SvO_2_ and nSsO_2_ decreased. The significantly reduced cardiac output (CO), MAP, and hemoglobin suggested an established low circulatory state (Table 1).

### The relationship between SQV/nSsO_2_ and SvO_2_

The conditional R^2^ for the mixed-effects model was 0.71, indicating that 71% of the variance in SvO_2_ was accounted for when including the random subject effects. The marginal R^2^ was 0.37, corresponding to the variance attributable to SQV independent of between-subject differences. Visual inspections of individual raw data, together with R^2^ advocated a linear relationship between changes in SQV and SvO_2_.

Using the mixed model's coefficients for the individual subjects (Table 2), we estimated nSsO_2_ according to:

**Table 2:** Individual regression coefficients from the mixed model

Pig	Intercept	Slope	R^2^
1	18.48	28.27	0.51
2	38.54	10.67	0.01
4	21.52	31.15	0.48
5	19.63	11.80	0.06
6	35.04	31.45	0.85
7	38.08	33.23	0.77
8	29.29	31.53	0.85
9	24.32	35.41	0.76
10	37.12	30.52	0.68
11	27.47	26.63	0.48
12	19.15	24.86	0.68
13	28.53	30.62	0.79
14	23.86	36.31	0.87
15	23.70	29.80	0.45

$${nSsO}_{2}=Intercept+slope\bullet SQV$$

**Fig. 2:**
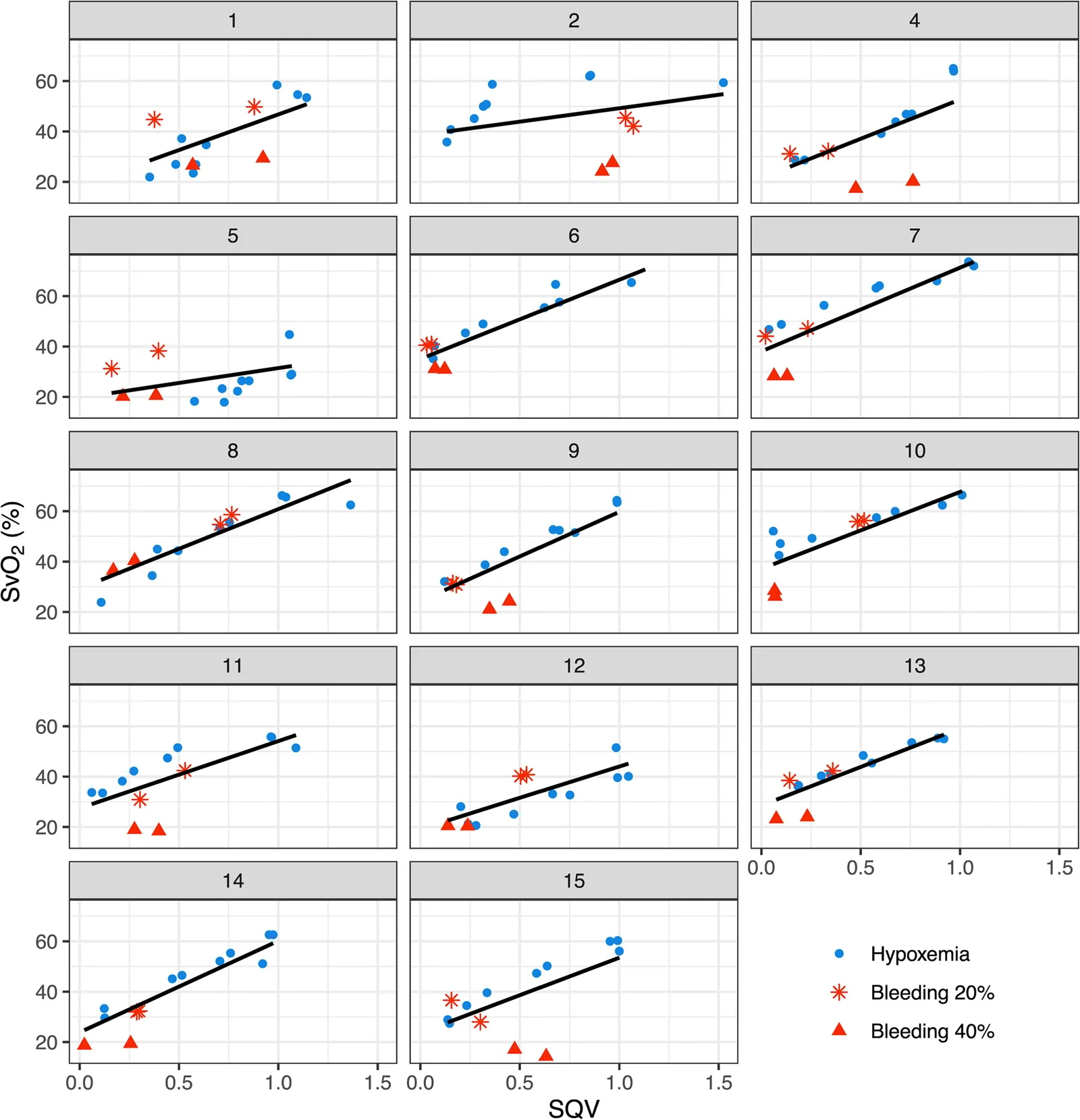
A scatterplot of raw data for mixed venous oxygen saturation (SvO_2_) and sternal probe quotient value (SQV) for each individual, together with a regression line illustrating individual estimates of non-invasive sternal intramedullary oxygen saturation (nSsO_2_) from the mixed model. For the hypovolemic data points, the red asterisks denote those originating from the 20% bleeding, and the triangles those from the 40% bleeding, respectively

Figure 2 shows the individual raw data of SQV and SvO_2_, together with a regression line illustrating the nSsO_2_ estimate.

To assess whether the slopes between the individuals were similar, a subanalysis was performed comparing two models with either a fixed or random slope. Allowing subject-specific slopes significantly improved model fit when all animals were included. However, inspection of the data revealed two animals (number 2 and 5) with markedly different slopes (Table 2, Fig. 2). After exclusion of these outliers, a random-slope model no longer improved model fit (likelihood ratio test χ^2^ = 0.03, *p* = 0.98), indicating that the remaining animals shared a common slope.

**Fig. 3:**
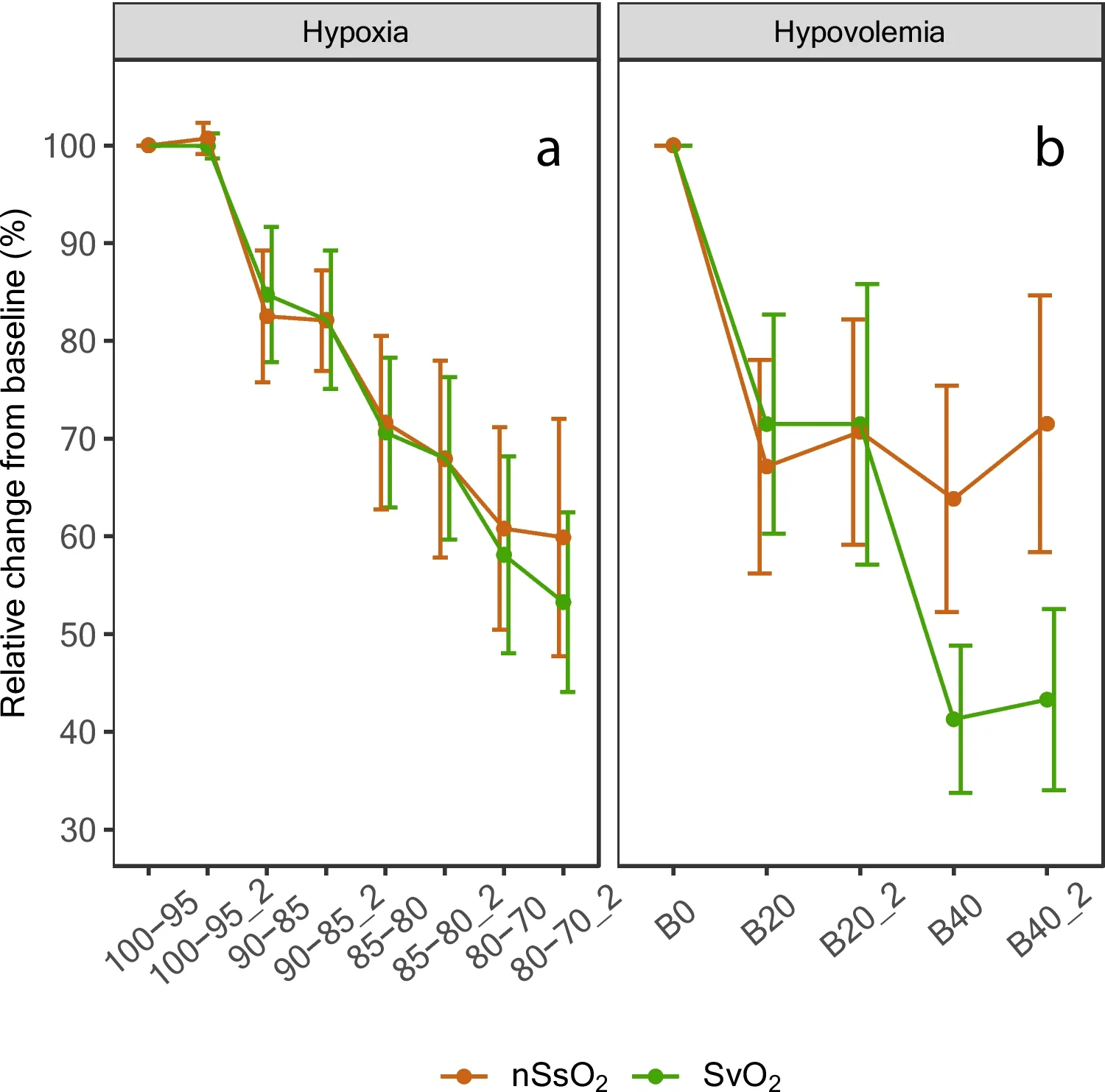
Relative change in percent from baseline for non-invasive sternal intramedullary oxygen saturation (nSsO_2_) and mixed venous oxygen saturation (SvO_2_) during the hypoxic (**a**) and hypovolemic (**b**) provocations, respectively. X-axis tics denote the first and second measurements for the various oxygen saturation plateaus (hypoxemia), and the percent bleeding (hypovolemia), respectively. Data is presented as mean ± SD

To compare the extent of change of either nSsO_2_ or SvO_2_ during the different provocations, relative changes, normalized to baseline values, are presented in Fig. 3.

### Trend analysis between nSsO_2_ and SvO_2_

**Fig. 4:**
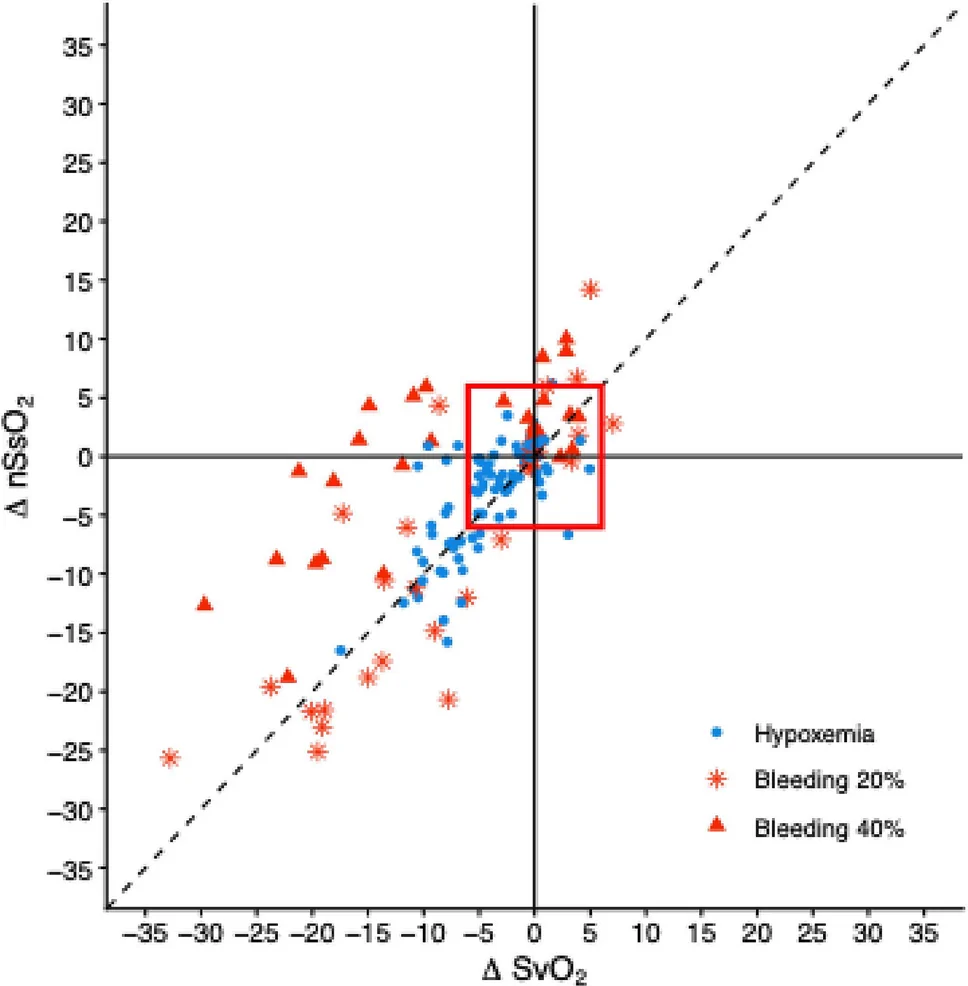
Four quadrant plot illustrating paired consecutive differences for mixed venous oxygen saturation (SvO2) and non-invasive sternal intramedullary oxygen saturation (nSsO2), respectively. Concordant data pairs are found in quadrant 1 (upper right) and 3 (lower left). The exclusion zone is highlighted by the central red box. Data points originating from the hypoxemic provocation are colored in blue, and those from the hypovolemic provocation in red, respectively. The red asterisks denote data points from the 20% bleeding level, and the triangles those from the 40% bleeding level, respectively

A total of 144 complete data pairs from the different provocations were available for concordance analysis. Of those, 76 data pairs had a consecutive difference of less than 6 oxygen percentage points, hence were within the exclusion zone. The remaining 68 data points (31 originating from the hypoxemic provocation, and 37 from the hypovolemic provocation, respectively) were available to assess trending (Fig. 4). From the four-quadrant plot the pooled concordance rate, for the two provocations, was 87% (95% CI 79.1–95.0). The concordance rates for the hypoxic and hypovolemic provocations were 90.6% (95% CI 80.5–100) and 83.8 (95% CI 71.9–95.7) respectively.

A post hoc sensitivity analysis including all 144 data points for the pooled concordance rate was performed, which demonstrated a concordance rate of 75.4% (95% CI 68.2–82.6). When studying Figs. 2, and 4, respectively it is evident that the data points originating from the 40% bleeding level often deviates from the rest. Therefore, an exploratory post hoc sensitivity analysis was performed excluding those data from the pooled concordance analysis. The resulting 51 data pairs demonstrated a pooled concordance rate of 92.2% (95% CI 84.8–99.6). 

## Discussion

In this porcine model, the main findings are that SQV, measured non-invasively on the sternum, and SvO_2_, determined by CO-oximetry, are linearly correlated on an individual subject level. This correlation can be used to estimate nSsO_2_, which co-varies with changes in SvO_2_ during hypoxemia, and mild to moderate hypovolemia.

The current study is a progression of a previous study, in which we demonstrated that SsO_2_ has more similarities with SvO_2_ than arterial oxygen saturation [11]. Our first objective in this study was to calibrate SQV measurements against SvO_2_. This calibration procedure is similar to the ones undertaken when developing new pulse oximeters, in the sense that a calibration curve is empirically obtained by measuring absorption quotients between red and infrared lights at a spectrum of varied oxygen saturations [23]. Usually, the pulse oximeter quotient formed is a ratio of AC- and DC-components to isolate the pulsatile component [23]. Our proposed method only utilizes the DC-components of red and infrared lights, thus determines the total change in absorbance when illuminating a tissue volume [10, 11]. To only account for DC-component alterations is an approach towards the NIRS-methodology. The feasibility of using DC-PPG in a NIRS-like manner has been shown by others [24].

Our rationale for assessing PPG on sternal blood flow are based on the facts that intramedullary PPG measurements are stable and less susceptible to external influence such as temperature or pressure [25, 26]. The sternum has a central anatomical location, supporting rapid detection of changes in oxygen saturation [10, 27, 28]. There are several arteries perfusing the bone, rendering PPG measurements stable [29, 30]. Also, the sternum is easily accessible.

Sternal PPG does not yield a direct measure of SvO_2_ but instead reflects regional sternal tissue oxygenation. Similarly, NIRS quantifies regional tissue oxygen saturation rather than true mixed venous oxygen saturation. Multiple anatomical measurement sites have been evaluated with NIRS to improve its agreement with SvO_2_ [3–7]. Since both PPG and NIRS are optically based, they share common sources for artifacts, such as shunting of light and susceptibility to motion. One distinction between the two, is that sternal PPG signals contain both the AC- and DC-components. Although, measurement of oxygenation is based on the DC-components, the preserved AC-components can be used for determination of pulse or respiratory rate [14, 15]. Unlike PPG, and NIRS, capnodynamics estimates SvO_2_ by analyzing the expiratory CO_2_ content, and has demonstrated promising agreement to measured SvO_2_. However, the technique is semi-invasive necessitating a mechanically ventilated patient [8].

A consequence of the automatic adjustment of signal quality was that the starting SQV became close to 1, regardless of SvO_2_. Hence, creating a population-level calibration curve was not feasible in this study since all pigs had slightly different SvO_2_ when the experiments commenced. Individual scatter plots of SQV and SvO_2_ demonstrated strong individual linear relationships, supported both by visual inspection, individual R^2^, and the conditional R^2^ of 0.71. Also, the slopes between individuals were found to be similar (Table 2, Fig. 2), except for a few individuals (subjects 2 and 5). For subject 2, all SQV-values for the hypovolemic provocation were high (Fig. 2). The recording from the sternal probe showed that the signal intensity of red light was higher than infrared, opposite to the hypoxic provocation in that animal, and the rest of the population (data not shown). The reason for this is unknown. For subject 5, obtaining a peripheral pulse oximeter reading was difficult due to peripheral vasoconstriction. The baseline cardiac output measurement was considerably lower than the rest of the pigs, and a proper separation of red and infrared light could not be seen on the sternal probe recordings from that individual.

During the hypoxic provocation (Fig. 3a), SvO_2_ and nSsO_2_ declined in parallel. However, during hypovolemia (Fig. 3b), SvO_2_ and nSsO_2_ only co-varied up to a moderate degree of hemorrhage (approximately 20%) [31], after which nSsO_2_ began to deviate from SvO_2_. During 40% volume loss, measured SQV, and consequently nSsO_2_ was higher than expected.

The divergence between nSsO_2_ and SvO_2_ observed during severe hypovolemia, has not been studied in detail, but may reflect fundamental differences between global and regional oxygenation measurements.

SvO_2_ represents the weighted average of all venous beds and is therefore highly sensitive to increases in systemic oxygen extraction. Experimental data demonstrate that rapid hemorrhage induces profound hemodynamic alterations and neurohumoral activation, resulting in marked increases in global oxygen extraction compared with graded bleeding [32]. As our model represents rapid hemorrhage, the pronounced decline in SvO_2_ (Fig. 3b) is consistent with such a systemic response.

In contrast, the sternal measurement reflects oxygenation within a localized bone marrow compartment. Since SaO_2_ remained constant throughout hypovolemia, the arteriovenous admixture may have attenuated the decline of nSsO_2_ compared with SvO_2_. Also, the sternum is highly vascularized and contains a sinusoidal microcirculation with relatively slow transit time and substantial intravascular volume, which might have reduced rapid fluctuations in oxygen saturation. Furthermore, due to its central anatomical location and crucial role in erythropoiesis, regional perfusion to the sternum may be relatively preserved during sympathetic redistribution of blood flow.

Methodological aspects of the DC-based optical approach may also be considered. Because nSsO_2_ is derived from non-pulsatile light attenuation components, its sensitivity depends on the fractional blood volume within the sampled tissue. During severe hypovolemia, a reduction in local blood volume may decrease the relative contribution of hemoglobin to the overall optical signal. As a consequence, further decreases in venous oxygen saturation may produce proportionally smaller changes in the DC ratio, resulting in a plateau-like behavior despite continued systemic desaturation [33].

Because different tissues have distinct metabolic rates and oxygen extraction patterns, the resulting venous oxygen saturation can vary between vascular beds. Consequently, a venous oxygen saturation measured in one compartment cannot be directly substituted for that from another site. This is particularly true, when comparing a local to global level of oxygen balance. Thus, SsO_2_/nSsO_2_ and SvO_2_ cannot be considered directly exchangeable. However, the measured SQV/SvO_2_-slopes between the individuals were similar. This suggests a uniform change in SQV for every alteration in SvO_2_ supporting the feasibility of continuous trend monitoring of SvO_2_ variations.

The pooled concordance from both provocations did not reach the desired threshold of 92%, although the result was within the 95% CI. The lower degree of concordance between nSsO_2_ and SvO_2_ during hypovolemia likely reduced the overall concordance rate. Thresholds for determining reliable trending have been proposed by studying different methods of cardiac output monitoring [19]. Whether those thresholds are the best for our proposed method is unknown. Despite that, we chose to adhere to the most described ones. Since the device is primarily intended as a trend monitor, and not to detect instant changes, we believe that a concordance level of 87% is sufficient for clinical purposes.

At current, the sternum probe has potential for continuous intra-individual monitoring of either increases or decreases of SvO_2_, possibly also with an indication of the magnitude change by analyzing the rate of change from the SQV/SvO_2_-slope. An individual single-point calibration of SvO_2_ from a blood sample is not regarded feasible. Future possibilities of creating a population level calibration curve would probably require modifying or removing the automatic calibration in its current form. It is also possible that, if the starting SQV could have been set freely, the marginal R^2^ might have increased substantially, since the different individuals shared a common slope. Due to different volumes of subcutaneous tissue, the between-subject variability may also have been influenced by variations in the amount of light reaching the sternal marrow. Increasing the penetration depth of light by moving the probe light source and photodetector even further apart, may help mitigate this effect.

Pulse oximeters sense the arterial oxygen saturation, which decreases when there is a reduction, or a mismatch in ventilation with pulmonary perfusion. Sternal PPG measurements could therefore have potential as a parallel complement to standard pulse oximetry. A decrease in both modalities would be consistent with hypoxic hypoxemia. However, if pulse oximetry remains stable while nSsO_2_ decreases, this may indicate increasing tissue oxygen extraction, for example due to hypovolemia.

This study failed to demonstrate a co-variation between nSsO_2_ and SvO_2_ for a 40% volume loss level. But such severe hypovolemia would have been clearly evident from basic circulatory monitoring. Early detection of disturbances in oxygen balance may be clinically relevant, which nSsO_2_ monitoring might provide.

### Limitations

This study has several limitations that need to be addressed. The study utilized pigs as subjects, and the results cannot be directly translated for human use. Despite the similarities in physiology between man and pigs [34], it is unknown how the oxygen balance in the human sternum varies with hypovolemia. Also, a mean weight of 33.1 kg implies young individuals with an uncertain ossification of the sternum as well as a high metabolic turnover and high baseline oxygen consumption, as seen from the relatively low baseline SvO_2_-values (Table 1).

The sternal probe is not explicitly designed for porcine use. The differences between pigs and humans regarding sternal width and segmentation, introduced potential inaccuracies in measuring intramedullary blood flow, and consequently, nSsO_2_-estimation. Moreover, some subjects had a variation of their surface anatomy, resembling Pectus excavatum. Although the LED-light and photodetector geometry entails deep light penetration, the increased amount of subcutaneous tissue in those individuals might have affected the measurements and increased the between-subject variability. Individual anatomical differences, such as skin thickness, pigmentation, sternal variations and perfusion, has the greatest impact on SQV values from the automatic calibration of the sternal probe. However, since the sternal probe had to be restarted between the provocations, an intra-individual discrepancy in SQV-calibration cannot be ruled out.

Muscle relaxation was used in order to reduce motion artifacts, still possible motion artifacts from the handling of the animal cannot be ruled out. Since the probe analyzes light reflection, it is dependent upon a good skin contact. Despite thorough skin preparation, the possibility for probe-skin interface issues with shunting of light and measurement interferences cannot be excluded.

Statistical limitations likely reflect both the modest sample size, especially after excluding the participant unable to complete the protocol, and the predefined exclusion zone in the concordance analysis, which eliminated a large proportion of observations. This may have resulted in an inflation of the concordance rate but also yielding wider confidence intervals and diminished power to identify genuine changes.

The current study has only evaluated the relationship between nSsO_2_ and SvO_2_ during hypoxemia and hypovolemia. However, other conditions affecting SvO_2_ exist, such as early sepsis, fever, pain/stress, and thyroid hormones [35]. How these situations affect SsO_2_ has not yet been clarified. Also, general anesthesia and neuromuscular blockade may affect SvO_2_ by altering oxygen consumption and cardiac output. How the current results of calibrating SQV to SvO_2_ can be translated to the awake individual, has not yet been investigated.

## Conclusions

This study has demonstrated the feasibility of calibrating non-invasive sternal PPG measurements against SvO_2_, with the intent to continuously monitor changes in global oxygen utilization.

Alterations in SvO_2_ during hypoxemia or mild/moderate hypovolemia may be tracked by sternal PPG-measurements, with high temporal resolution. However, further technical development of the current prototype probe is necessary to allow for inter-individual comparisons of nSsO_2_.

## Data Availability

The datasets used and/or analyzed during the current study are available from the corresponding author on reasonable request.

## References

[1] Bussières JS (2007) Iatrogenic pulmonary artery rupture. Curr Opin Anesthesiol 20:48. 10.1097/ACO.0b013e32801158a9 17211167

[2] Vincent J-L (2012) The pulmonary artery catheter. J Clin Monit Comput 26:341–345. 10.1007/s10877-012-9389-2 22886686

[3] Guan Z, Liu J-J, Yuan H, Gao Y-F (2018) Positive correlation between regional cerebral oxygen saturation and mixed venous oxygen saturation during off-pump coronary artery bypass surgery. Respir Care 63:988–993. 10.4187/respcare.05772 29844213

[4] Moerman A, Vandenplas G, Bové T et al (2013) Relation between mixed venous oxygen saturation and cerebral oxygen saturation measured by absolute and relative near-infrared spectroscopy during off-pump coronary artery bypass grafting. Br J Anaesth 110:258–265. 10.1093/bja/aes375 23103778

[5] Schön J, Heringlake M, Berger K-U et al (2011) Relationship between mixed venous oxygen saturation and regional cerebral oxygenation in awake, spontaneously breathing cardiac surgery patients. Minerva Anestesiol 77:952–958 21952594

[6] Tortoriello TA, Stayer SA, Mott AR et al (2005) A noninvasive estimation of mixed venous oxygen saturation using near-infrared spectroscopy by cerebral oximetry in pediatric cardiac surgery patients. Paediatr Anaesth 15:495–503. 10.1111/j.1460-9592.2005.01488.x 15910351

[7] Jeleff A, Suh N, Schranc Á et al (2024) New noninvasive method for the assessment of central venous oxygen saturations in critically ill patients. J Cardiothorac Vasc Anesth 38:170–174. 10.1053/j.jvca.2023.09.020 37827917

[8] Karlsson J, Lönnqvist PA, Wallin M, Hallbäck M (2021) A continuous noninvasive method to assess mixed venous oxygen saturation: a proof-of-concept study in pigs. Anesth Analg 132:1768–1776. 10.1213/ane.0000000000005188 33009138

[9] Dunn J-O, Mythen M, Grocott M (2016) Physiology of oxygen transport. BJA Educ 16:341–348. 10.1093/bjaed/mkw012

[10] Näslund E, Lindberg LG, Lund I et al (2020) Measuring arterial oxygen saturation from an intraosseous photoplethysmographic signal derived from the sternum. J Clin Monit Comput 34:55–62. 10.1007/s10877-019-00289-w PMC6946764 30805761

[11] Näslund E, Lindberg L-G, Strandberg G et al (2023) Oxygen saturation in intraosseous sternal blood measured by CO-oximetry and evaluated non-invasively during hypovolaemia and hypoxia - a porcine experimental study. J Clin Monit Comput 37:847–856. 10.1007/s10877-023-00980-z PMC10175432 36786963

[12] Farzam P, Lindner C, Weigel UM et al (2014) Noninvasive characterization of the healthy human manubrium using diffuse optical spectroscopies. Physiol Meas 35:1469–1491. 10.1088/0967-3334/35/7/1469 24901605

[13] Schwartz BM, Stats D (1949) Oxygen saturation of sternal marrow blood in polycythemia Vera. J Clin Invest 28:736–740. 10.1172/jci102125 PMC439656 16695732

[14] Henricson J, Glasin J, Rindebratt S, Wilhelms D (2022) Respiratory rate monitoring in healthy volunteers by central photoplethysmography compared to capnography. J Biophotonics 15:e202100270. 10.1002/jbio.202100270 34874126

[15] Glasin J, Henricson J, Lindberg LG, Wilhelms D (2019) Wireless vitals-proof of concept for wireless patient monitoring in an emergency department setting. J Biophotonics 12:e201800275. 10.1002/jbio.201800275 30306737

[16] Nitzan M, Nitzan I, Arieli Y (2020) The various oximetric techniques used for the evaluation of blood oxygenation. Sens Basel 20:4844. 10.3390/s20174844 PMC7506757 32867184

[17] Meteyard L, Davies RAI (2020) Best practice guidance for linear mixed-effects models in psychological science. J Mem Lang 112:104092. 10.1016/j.jml.2020.104092

[18] Nakagawa S, Schielzeth H (2013) A general and simple method for obtaining R2 from generalized linear mixed-effects models. Methods Ecol Evol 4:133–142. 10.1111/j.2041-210x.2012.00261.x

[19] Critchley LA, Lee A, Ho AM-H (2010) A critical review of the ability of continuous cardiac output monitors to measure trends in cardiac output. Anesth Analg 111:1180. 10.1213/ANE.0b013e3181f08a5b 20736431

[20] Svedmyr A, Konrad M, Wallin M et al (2022) Non-invasive capnodynamic mixed venous oxygen saturation during major changes in oxygen delivery. J Clin Monit Comput 36:1315–1324. 10.1007/s10877-021-00762-5 PMC8490846 34609659

[21] Cecconi M, Rhodes A, Poloniecki J et al (2009) Bench-to-bedside review: the importance of the precision of the reference technique in method comparison studies – with specific reference to the measurement of cardiac output. Crit Care 13:201. 10.1186/cc7129 PMC2688094 19183431

[22] Axiak Flammer SM, Critchley LA, Weber A et al (2013) Reliability of lithium dilution cardiac output in anaesthetized sheep. Br J Anaesth 111:833–839. 10.1093/bja/aet220 23838805

[23] Chan ED, Chan MM, Chan MM (2013) Pulse oximetry: understanding its basic principles facilitates appreciation of its limitations. Respir Med 107:789–799. 10.1016/j.rmed.2013.02.004 23490227

[24] Abay TY, Kyriacou PA (2018) Photoplethysmography for blood volumes and oxygenation changes during intermittent vascular occlusions. J Clin Monit Comput 32:447–455. 10.1007/s10877-017-0030-2 PMC5943414 28547651

[25] Näslund J, Pettersson J, Lundeberg T et al (2006) Non-invasive continuous estimation of blood flow changes in human patellar bone. Med Biol Eng Comput 44:501–509. 10.1007/s11517-006-0070-0 16937201

[26] Mateus J, Hargens AR (2012) Photoplethysmography for non-invasive in vivo measurement of bone hemodynamics. Physiol Meas 33:1027–1042. 10.1088/0967-3334/33/6/1027 22562998

[27] Mannheimer PD, Bebout DE (2002) The OxiMax System. Nellcor’s new platform for pulse oximetry. Minerva Anestesiol 68:236–239 12024089

[28] Lindholm P, Blogg SL, Gennser M (2007) Pulse oximetry to detect hypoxemia during apnea: comparison of finger and ear probes. Aviat Space Env Med 78:770–773 17760284

[29] Berdajs D, Zund G, Turina MI, Genoni M (2006) Blood supply of the sternum and its importance in internal thoracic artery harvesting. Ann Thorac Surg 81:2155–2159. 10.1016/j.athoracsur.2006.01.020 16731146

[30] de Jesus RA, Acland RD (1995) Anatomic study of the collateral blood supply of the sternum. Ann Thorac Surg 59:163–168. 10.1016/0003-4975(94)00722-j 7818316

[31] American College of Surgeons (2012) Advanced trauma life support: ATLS ; student course manual, 9. ed. American College of Surgeons, Chicago, Ill

[32] Dyson A, Ekbal N, Stotz M et al (2014) Component reductions in oxygen delivery generate variable haemodynamic and stress hormone responses. Br J Anaesth 113:708–716. 10.1093/bja/aeu089 24852502

[33] Allen J (2007) Photoplethysmography and its application in clinical physiological measurement. Physiol Meas 28:R1-39. 10.1088/0967-3334/28/3/r01 17322588

[34] Swindle MM, Makin A, Herron AJ et al (2012) Swine as models in biomedical research and toxicology testing. Vet Pathol 49:344–356. 10.1177/0300985811402846 21441112

[35] Hartog C, Bloos F (2014) Venous oxygen saturation. Best Pract Res Clin Anaesthesiol 28:419–428. 10.1016/j.bpa.2014.09.006 25480771

